# Involvement of Circulating Exosomal MicroRNAs in Jian-Pi-Yi-Shen Formula Protection Against Adenine-Induced Chronic Kidney Disease

**DOI:** 10.3389/fphar.2020.622658

**Published:** 2021-02-02

**Authors:** Xinhui Liu, Siqi Liu, Denggui Luo, Shiying Huang, Fochang Wang, Bing Zhang, Yulian Chen, Lin Zheng, Jiandong Lu, Shunmin Li

**Affiliations:** ^1^ Department of Nephrology, Shenzhen Traditional Chinese Medicine Hospital, Guangzhou University of Chinese Medicine, Shenzhen, China; ^2^ Shenzhen Key Laboratory of Hospital Chinese Medicine Preparation, Shenzhen Traditional Chinese Medicine Hospital, Guangzhou University of Chinese Medicine, Shenzhen, China

**Keywords:** chronic kidney disease, traditional Chinese medicine, Jian-Pi-Yi-Shen formula, exosomal microRNAs, small RNA sequencing

## Abstract

Jian-Pi-Yi-Shen formula (JPYSF) is a traditional Chinese medicine (TCM) formula used in clinic to treat chronic kidney disease (CKD) for decades. However, the mechanisms of JPYSF in treating CKD have not been fully elucidated. The aim of the present study was to test the renoprotective effect of JPYSF on CKD rat model and investigate the potential mechanism from the perspective of serum exosomal microRNAs (miRNAs). CKD rat model was induced by feeding Sprague-Dawley rats a diet containing 0.75% *w/w* adenine for four weeks. The rats in the treatment group were given 10.89 g/kg JPYSF by gavage every day, starting from the 3rd week of the adenine-containing diet for six weeks. Serum biochemistry and histopathology were used to evaluate the renoprotective effects of JPYSF. Serum exosomes were isolated by ExoQuick-TC PLUS exosomes extraction kit and were identified by transmission electron microscopy, nanoparticle tracking analysis, and western blot. Exosomal miRNAs profiling was analyzed by small RNA sequencing. The results showed that JPYSF treatment significantly lowered serum creatinine and blood urea nitrogen levels and alleviated renal pathological injury in CKD rats. Furthermore, serum exosomes were successfully isolated and identified. Small RNA sequencing revealed that 4 exosomal miRNAs (miR-192-5p, miR-194-5p, miR-802-5p, and miR-143-3p) were significantly downregulated in the CKD group and were markedly upregulated after JPYSF treatment. At last, miR-192-5p was identified as the most relevant miRNA for CKD diagnosis and JPYSF treatment. In conclusion, JPYSF protects kidney from adenine-induced CKD, which may be associated with modulation of exosomal miRNAs.

## Introduction

According to the data reported by the Global Burden of Disease Study, in 2017, 697.5 million cases of all-stage chronic kidney disease (CKD) were recorded, for a global prevalence of 9.1%. Globally, in 2017, 1.2 million (95% uncertainty interval, 1.2 to 1.3) people died from CKD (Bikbov et al., 2020). CKD is an important contributor to morbidity and mortality and has also been recognized as a risk factor for cardiovascular disease (Sarnak et al., 2003). The exploration of treatment strategies and associated mechanism investigation are therefore highly desirable to halt CKD progression. The Jian-Pi-Yi-Shen formula (JPYSF) is a traditional Chinese medicine (TCM) formula that has a clinical application in the treatment of CKD with notable renoprotective effect. Our previous pharmacological studies have also showed the renoprotective effect of JPYSF in CKD rat model (Liu et al., 2018b; Lu et al., 2018). However, the underlying mechanisms of JPYSF on CKD have not been fully elucidated.

Recently, exosomes have drawn considerable attentions in renal diseases for their pathophysiologic, diagnostic, and therapeutic roles (Krause et al., 2015; Pomatto et al., 2017). Exosomes are generated from multivesicular bodies with sizes between 30 and 100 nm in diameter (Karpman et al., 2017). Exosomes can be released by various types of cells into blood or other body fluids under physiologic and pathologic conditions, which reflects the cellular response to internal and external environment (Lv et al., 2019; Tang et al., 2019). The cargoes in exosomes include proteins, microRNAs (miRNAs), long noncoding RNAs, mRNAs, and lipids (Kim et al., 2015). miRNAs are noncoding, single-stranded small RNAs that modulate gene expression through binding to the 3′-untranslated regions (3′-UTRs) of target mRNAs for degradation or translational repression (Bartel, 2009). Recent studies found that miRNAs are commonly enriched in exosomes (Squadrito et al., 2014) and there are several advantages of exosomal miRNAs, such as remarkable stability, resistance to degradation, and reflection of pathophysiological processes (Bala et al., 2012). Hence, investigating exosomal miRNAs provides a useful platform to understand the mechanisms and progresses of diseases. It has been reported that exosomal miRNA-19b-3p and miRNA-23a were involved in tubulointerstitial inflammation in lipopolysaccharide induced acute kidney injury model (Lv et al., 2020) and ischemia/reperfusion induced kidney injury model (Li et al., 2019), respectively. Lv et al. found that urinary exosomal miR-29c correlated with both renal function and degree of histological fibrosis in CKD patients (Lv et al., 2013). The role of exosomal miRNAs in kidney disease is gaining more and more attention. Whether the renoprotective effect of JPYSF in the treatment of CKD is related to the regulation of exosomal miRNAs is currently unknown.

In the present study, we tested the renoprotective effect of JPYSF on adenine-induced CKD rat model by using renal function and renal pathology. Moreover, circulating exosomes were isolated from serum samples and small RNA sequencing was applied to investigate the exosomal miRNAs profiling in CKD rats with or without JPYSF treatment.

## Materials and Methods

### Preparation of JPYSF

JPYSF is composed of eight herbs: Astragali Radix, Atractylodis Macrocephalae Rhizoma, Dioscoreae Rhizoma, Cistanches Herba, Amomi Fructus Rotundus, Salviae Miltiorrhizae Radix et Rhizoma, Rhei Radix et Rhizoma, and Glycyrrhizae Radix et Rhizoma Praeparata cum Melle (Table 1). The plant names have been validated with http://mpns.kew.org/mpns-portal/?_ga=1.111763972.1427522246.1459077346. The herbs were purchased from Shenzhen Huahui Pharmaceutical Co., Ltd. (Shenzhen, China). They were authenticated by Professor Shangbin Zhang, Department of Pharmacy, Shenzhen Traditional Chinese Medicine Hospital. Their voucher specimens were deposited at Shenzhen Traditional Chinese Medicine Hospital. These raw herbs were processed into lyophilized powder and then high-performance liquid chromatography–mass spectrometry analysis was conducted to confirm the quality of JPYSF as previously described (Liu et al., 2018b).

**TABLE 1:** Composition of Jian-Pi-Yi-Shen formula (JPYSF).

Plant name	Herb name	Chinese name	Medicinal part	Voucher number	Dosage (g)
*Astragalus membranaceus* (Fisch.) Bge. var. *mongholicus* (Bge.) Hsiao	Astragali Radix	Huang Qi	Root	2010015Z	30
*Atractylodes macrocephala* Koidz.	Atractylodis Macrocephalae Rhizoma	Bai Zhu	Rhizome	2010024ZZ	10
*Dioscorea opposita* Thunb.	Dioscoreae Rhizoma	Shan Yao	Rhizome	2010037Z	30
*Cistanche deserticola* Y.C. Ma	Cistanches Herba	Rou Cong Rong	Fleshy stem	2040056Z	10
*Amomum kravanh* Pierre ex Gagnep.	Amomi Fructus Rotundus	Dou Kou	Fruit	202086Z	10
*Salvia miltiorrhiza* Bunge.	Salviae Miltiorrhizae Radix et Rhizoma	Dan Shen	Root and Rhizomes	2010006Z	15
*Rheum palmatum* L.	Rhei Radix et Rhizoma	Da Huang	Root and Rhizomes	2010040Z	10
*Glycyrrhiza uralensis* Fisch.	Glycyrrhizae Radix et Rhizoma Praeparata cum Melle	Zhi Gan Cao	Root and Rhizomes	2010008ZZ	6

### Animals

Healthy male Sprague-Dawley (SD) rats weighting 150–180 g were purchased from Guangdong Medical Laboratory Animal Center (Foshan, China). All animal experimental protocols were approved by the Experimental Animal Ethics Committee of Guangzhou University of Chinese Medicine. After one week of acclimatization, all rats were randomly divided into the following three groups (n = 6 rats per group): (i) control group; (ii) CKD group; and (iii) JPYSF treatment group (CKD + JPYSF). CKD was induced by feeding a diet containing 0.75% *w/w* adenine (Sangon Biotech, Shanghai, China) for four weeks (Liu et al., 2019). The rats in the CKD + JPYSF group were given 10.89 g/kg JPYSF by gavage every day, starting from the 3rd week of the adenine-containing diet for six weeks. The rats in the control and CKD group were treated with equal volumes of distilled water. At the end of experiments, blood and kidney samples were rapidly collected and processed for further analysis.

### Serum Biochemistry

Serum samples were obtained from blood by centrifugation for 15 min at 2,000 × g at 4°C. Serum creatinine (Scr) and blood urea nitrogen (BUN) were tested by using Creatinine Serum Detection Kit and Blood Urea Nitrogen Detection Kit (StressMarq Biosciences, British Columbia, Canada), respectively, following the manufacturer’s instructions.

### Histopathology

Rat kidney tissues were fixed with 10% formalin at 4°C overnight, dehydrated with graded ethanol and embedded in paraffin. The paraffin-embedded kidneys were cut into 4 μm sections and stained with periodic acid–Schiff (PAS) and Masson’s trichrome stains to evaluate pathologic changes. Slides were viewed by Nikon microscope and representative pictures were captured by NIS-Elements BR software version 4.10.00 (Nikon, Japan). For semiquantitative analysis, the tubular atrophy in PAS staining was assessed as the following score: 0, normal tubules; 1, rare single atrophic tubule; 2, several clusters of atrophic tubules; and 3, massive atrophy. Fibrotic area in Masson staining was measured by using ImageJ software (NIH, Bethesda, MD, USA). Five microscopic fields (200×) of each rat and six rats in each group were used for quantitative analyses in a blinded manner.

### Serum Exosomes Isolation

Rat serum (1 mL) was centrifugated at 2,000 × g for 10 min at 4°C and then the supernatant was collected and centrifugated at 10,000 × g for 20 min at 4°C. After the supernatant was collected, exosomes were extracted by using ExoQuick-TC PLUS exosomes extraction kit (SBI System Biosciences, Palo Alto, CA, USA) according to the manufacturer’s protocol. The isolated exosomes were resuspended using 1 × phosphate buffered saline (PBS).

### Transmission Electron Microscopy

For conventional transmission electron microscopy (TEM), the exosome pellet was placed in a droplet of 2.5% glutaraldehyde in PBS and fixed. Samples were rinsed and postfixed in 1% osmium tetroxide. These samples were embedded in 10% gelatine, fixed and cut into several blocks (<1 mm^3^). The samples were dehydrated in increasing concentrations of alcohol and infiltrated with increasing concentrations of Quetol-812 epoxy resin mixed with propylene oxide. Samples were embedded in pure fresh Quetol-812 epoxy resin and polymerized. Ultrathin sections (100 nm) were cut by using a Leica UC6 ultramicrotome and post-stained with uranyl acetate for 10 min and lead citrate for 5 min at room temperature, followed by observation with a Hitachi H-600 transmission electron microscope (Tokyo, Japan), operated at 120 kV.

### Nanoparticle Tracking Analysis

The exosome particles were sized and enumerated by nanoparticle tracking analysis (NTA) using a NanoSight 300 instrument (NanoSight Ltd, UK) equipped with NTA 3.2 software. NTA is a light-scattering technique that uses video analysis for the sizing and enumeration of extracellular vesicles. Exosomes samples were collected and diluted in PBS to a particle concentration within the range of 2–20 × 10^8^/mL (optimal working range of the system). An approximately 100 µL sample was loaded into the sample chamber, and 60 s videos were recorded for each sample with a shutter speed of approximately 30 ms and a camera gain between 250 and 650. The settings for software analysis were as follows: detection threshold, 30–50; blur, 535; and minimum expected particle size, auto.

### Western Blot

Samples were lysed with sodium dodecyl sulfate (SDS) sample buffer. Protein concentrations were determined using a BCA protein assay kit (Sigma-Aldrich, St Louis, MO, USA), and lysates were mixed with 4 × SDS loading buffer. Samples were heated at 100 °C for 5 min before loading and separated on precast 10% SDS-polyacrylamide gels (Bio-Rad Laboratories, Hercules, CA, USA). The detection of protein expression by western blot was performed as described previously (Liu et al., 2020). The primary antibodies were anti-CD9 (GeneTex, GTX76184), anti-CD81 (GeneTex, GTX101766), and antitumor susceptibility gene 101 protein (TSG101) (GeneTex, GTX70255).

### RNA Extraction

Total RNA was extracted from the exosomes using TRIzol® Reagent according the manufacturer’s instructions (Invitrogen, Carlsbad, CA, USA) and genomic DNA was removed using DNase I (TaKara, Tokyo, Japan). Then RNA quality was determined by 2100 Bioanalyzer (Agilent Technologies, Santa Clara, CA, USA) and quantified using the ND-2000 (NanoDrop Technologies, DE, USA). Only high-quality RNA sample (OD260/280 = 1.8∼2.2, OD260/230 ≥ 2.0, RIN ≥ 6.5, 28S:18S ≥ 1.0, >10 μg) was used to construct sequencing library.

### Library Preparation and Illumina Sequencing

RNA-seq transcriptome library was prepared following TruSeq RNA sample preparation kit from Illumina (San Diego, CA, USA) using 5 μg of total RNA. Shortly, messenger RNA was isolated according to poly-A selection method by oligo (dT) beads and then fragmented by fragmentation buffer firstly. Secondly double-stranded cDNA was synthesized using a SuperScript Double-Stranded cDNA Synthesis Kit (Invitrogen, CA, USA) with random hexamer primers (Illumina). Then the synthesized cDNA was subjected to end-repair, phosphorylation, and ‘A’ base addition according to Illumina’s library construction protocol. Libraries were size selected for cDNA target fragments of 200–300 bp on 2% Low Range Ultra Agarose followed by PCR amplified using Phusion DNA polymerase (NEB) for 15 PCR cycles. After being quantified by TBS380, paired-end RNA-seq sequencing library was sequenced with the Illumina HiSeq SE50.

### Read Mapping

The raw paired end reads were trimmed and quality controlled by SeqPrep (https://github.com/jstjohn/SeqPrep) and Sickle (https://github.com/najoshi/sickle) with default parameters. Then clean reads were separately aligned to reference genome with orientation mode using TopHat (http://tophat.cbcb.umd.edu, version2.0.0) software (Trapnell et al., 2009). The mapping criteria of bowtie were as follows: sequencing reads should be uniquely matched to the genome allowing up to two mismatches, without insertions or deletions. Then the regions of gene were expanded following depths of sites and the operon was obtained. In addition, the whole genome was split into multiple 15 kbp windows that share 5 kbp. New transcribed regions were defined as more than 2 consecutive windows without overlapped region of gene, where at least two reads mapped per window in the same orientation.

### Differential Expression Analysis and Functional Enrichment

To identify differential expression genes (DEGs) between two different samples, the expression level of each transcript was calculated according to the fragments per kilobase of exon per million mapped reads (FRKM) method. RSEM (http://deweylab.biostat.wisc.edu/rsem/) was used to quantify gene abundances (Li and Dewey, 2011). R statistical package software edgeR (Empirical analysis of Digital Gene Expression in R, http://www.bioconductor.org/packages/2.12/bioc/html/edgeR.html) was utilized for differential expression analysis (Robinson et al., 2010). In addition, functional-enrichment analyses including Gene Ontology (GO) and the Kyoto Encyclopedia of Genes and Genomes (KEGG) were performed to identify which DEGs were significantly enriched in GO terms and metabolic pathways at Bonferroni-corrected *p*-value ≤ 0.05 compared with the whole-transcriptome background. GO functional enrichment and KEGG pathway analysis were carried out by GOATOOLS (https://github.com/tanghaibao/Goatools) and KOBAS (http://kobas.cbi.pku.edu.cn/home.do) (Xie et al., 2011).

### Real-Time Quantitative PCR

Total RNA was isolated from kidney tissues by RNAiso Plus reagent (TaKaRa) according to the manufacturer’s instructions. cDNA template was prepared by using a miRcute Plus miRNA First-Strand cDNA Kit (TIANGEN Biotech, Beijing, China). Real-time quantitative PCR was performed using HieffTM qPCR SYBR Green Master Mix (YEASEN Biotech, Shanghai, China) on Applied Biosystems ABI Viia7 (Applied Biosystems, Foster City, CA, USA). The primers sequences were list in Table 2. The cycling condition was 95°C for 5min, followed by 40 repeats of 95°C for 10s and 60°C for 30s. The relative expression was calculated using the comparative threshold cycle values method (2^−ΔΔCt^) and normalized to the expression of small nuclear RNA U6.

**TABLE 2:** Sequence of the primers for real-time quantitative PCR.

Name	Primer sequence
Rno-miR-192-5p	CTG​ACC​TAT​GAA​TTG​ACA​GCC
Rno-miR-194-5p	GGT​GTA​ACA​GCA​ACT​CCA​TG
Rno-miR-802-5p	CAGTAACAAAGATTCATC
Rno-miR-143-3p	TGA​GAT​GAA​GCA​CTG​TAG​CTC​A
U6	GGA​ACG​ATA​CAG​AGA​AGA​TTA​G

### Statistical Analysis

Statistical analysis and box-and-whisker diagram establishment were performed by GraphPad Prism7.04 (La Jolla, CA, USA). Statistical differences were examined by one-way ANOVA followed by Tukey post hoc test. A value of *p* < 0.05 indicated statistically significant differences.

## Results

### JPYSF Exerted Renoprotective Effect on CKD Rats

**FIGURE 1:**
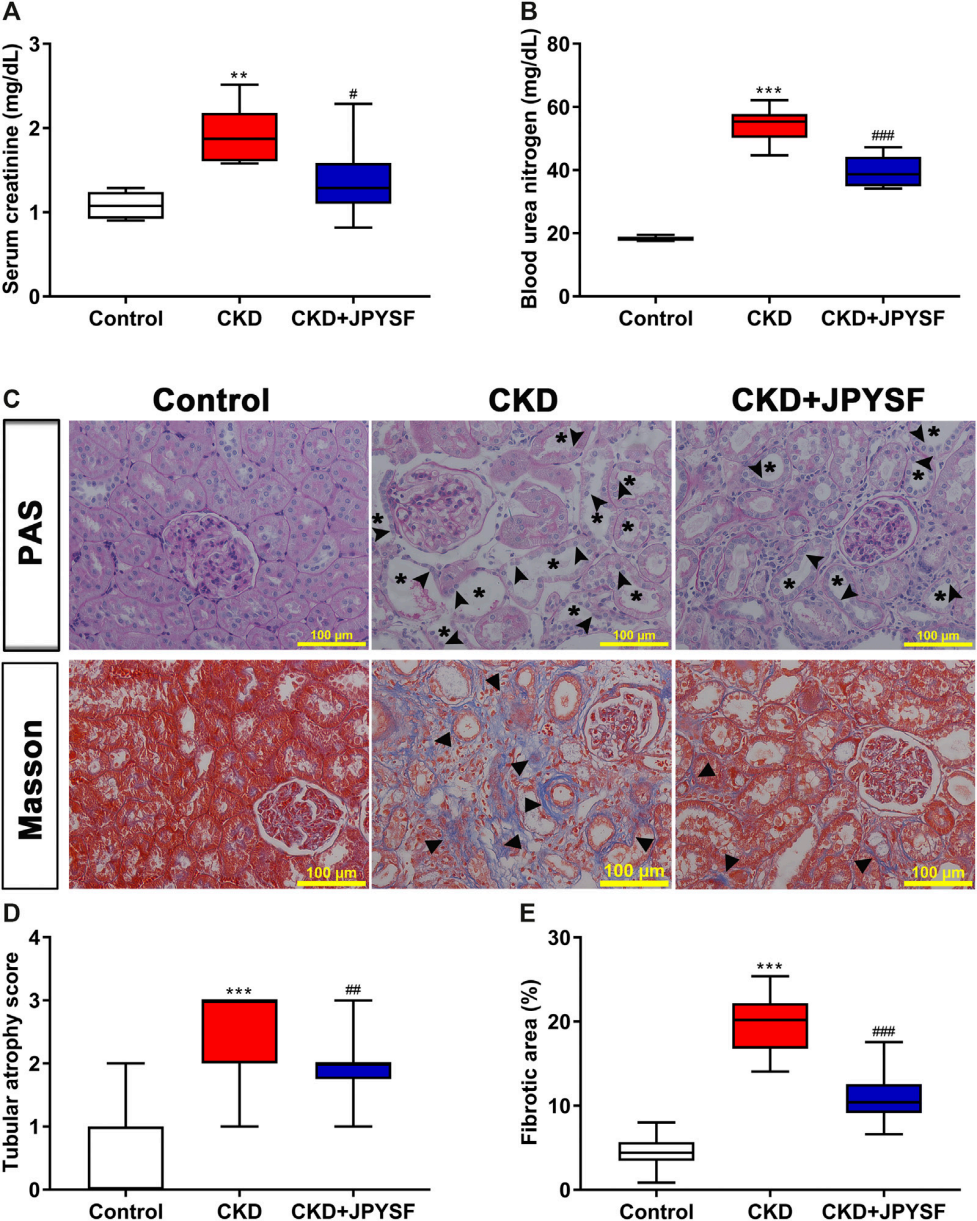
Effects of JPYSF on adenine-induced CKD rats. **(A)** Serum creatinine. **(B)** Blood urea nitrogen. **(C)** PAS and Masson staining. All images are shown at identical magnification, ×200, scale bar = 100 μm. Asterisk indicates tubular expansion, arrowhead indicates atrophic tubular epithelial cell, and triangle indicates collagen fibril. **(D)** Semiquantitative analysis of tubular atrophy in PAS staining. **(E)** Semiquantitative analysis of fibrotic area in Masson staining. n = 6 rats per group (**P < 0.01, ***P < 0.001 compared with the control group; ^#^ *P* < 0.05, ^##^ *P* < 0.01, ^###^ *P* < 0.001 compared with the CKD group).

Compared with the control group, the levels of Scr and BUN in the CKD group increased significantly. JPYSF treatment could reduce the Scr and BUN levels of CKD rats by 29% (*p* < 0.05) and 27% (*p* < 0.001), respectively (Figures 1A,B). Parallel to the deterioration of renal function, histopathological evaluation in the CKD group displayed massive tubular epithelial cells atrophy and tubular expansion in PAS staining and obvious accumulation of collagen fibrils (blue staining) in Masson staining. Consistent with the improvement of renal function, these pathological injuries were markedly attenuated in the CKD + JPYSF group (Figures 1C–E). These data demonstrated that JPYSF has a renoprotective effect in adenine-induced CKD rats.

### Characterization of Serum Exosomes

**FIGURE 2:**
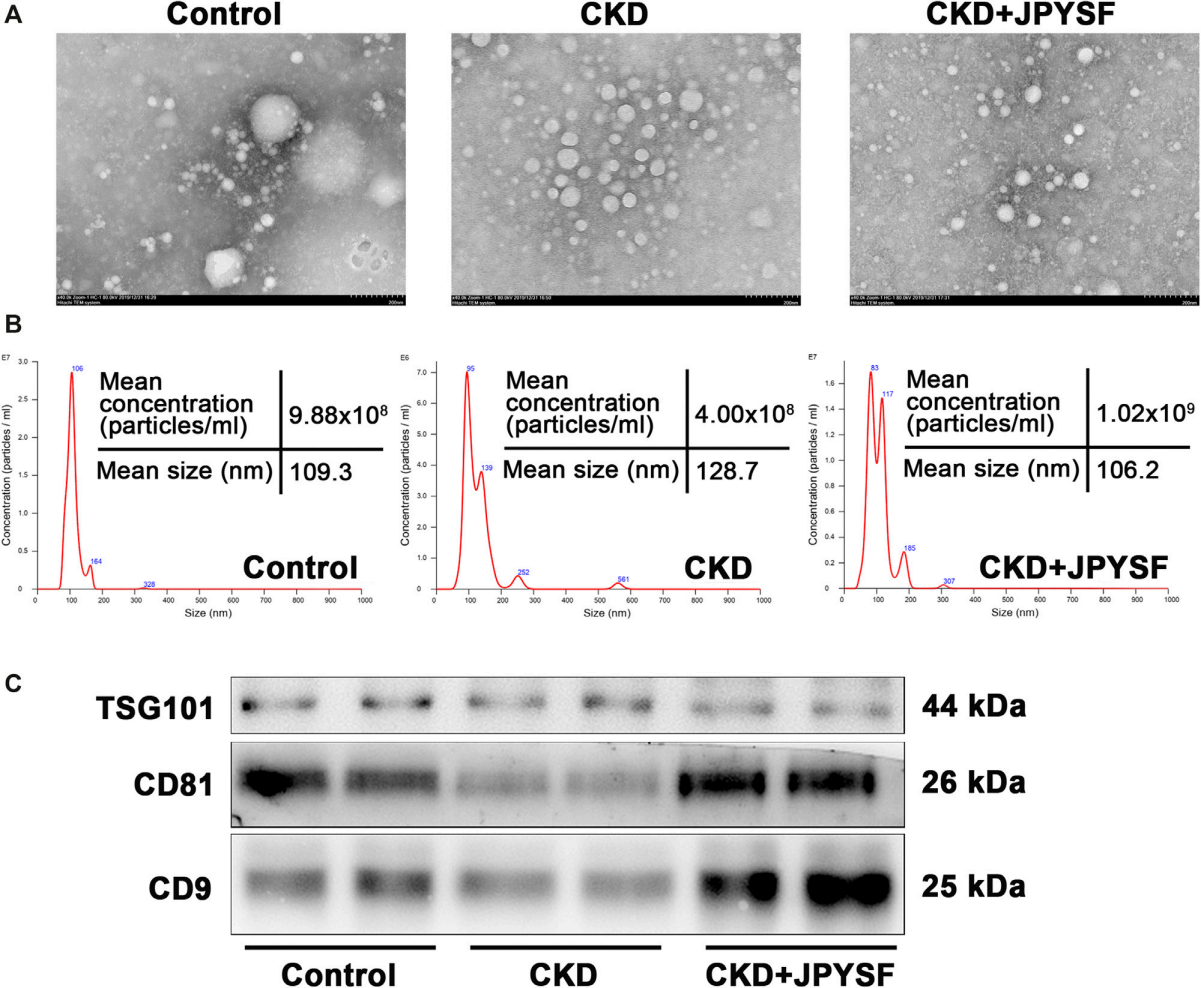
Identification of isolated serum exosomes. **(A)** Transmission electron microscope image of isolated serum exosomes. **(B)** NanoSight analysis of isolated serum exosomes. The horizontal axis represents particle size (nm), and the vertical axis represents particle concentration (particles/ml). **(C)** Western blot analysis of the expression of exosomal specific markers TSG101, CD81, and CD9.

The morphology, size distribution, and biomarkers expression were used to identify the characteristics of isolated serum exosomes. TEM showed the particles were spherical structures with diameter of approximately 100 nm (Figure 2A). NanoSight analysis revealed that the size of isolated particles ranged from 50 nm to 150 nm in diameter, consistent with the characteristic size range of exosomes (Figure 2B). In addition, western blot analysis indicated the presence of exosomal specific markers TSG101, CD81, and CD9 in isolated particles (Figure 2C). Taken together, these data indicated that circulating exosomes were successfully isolated from serum.

### Overview of Small RNA Sequencing in Serum Exosomes

**FIGURE 3:**
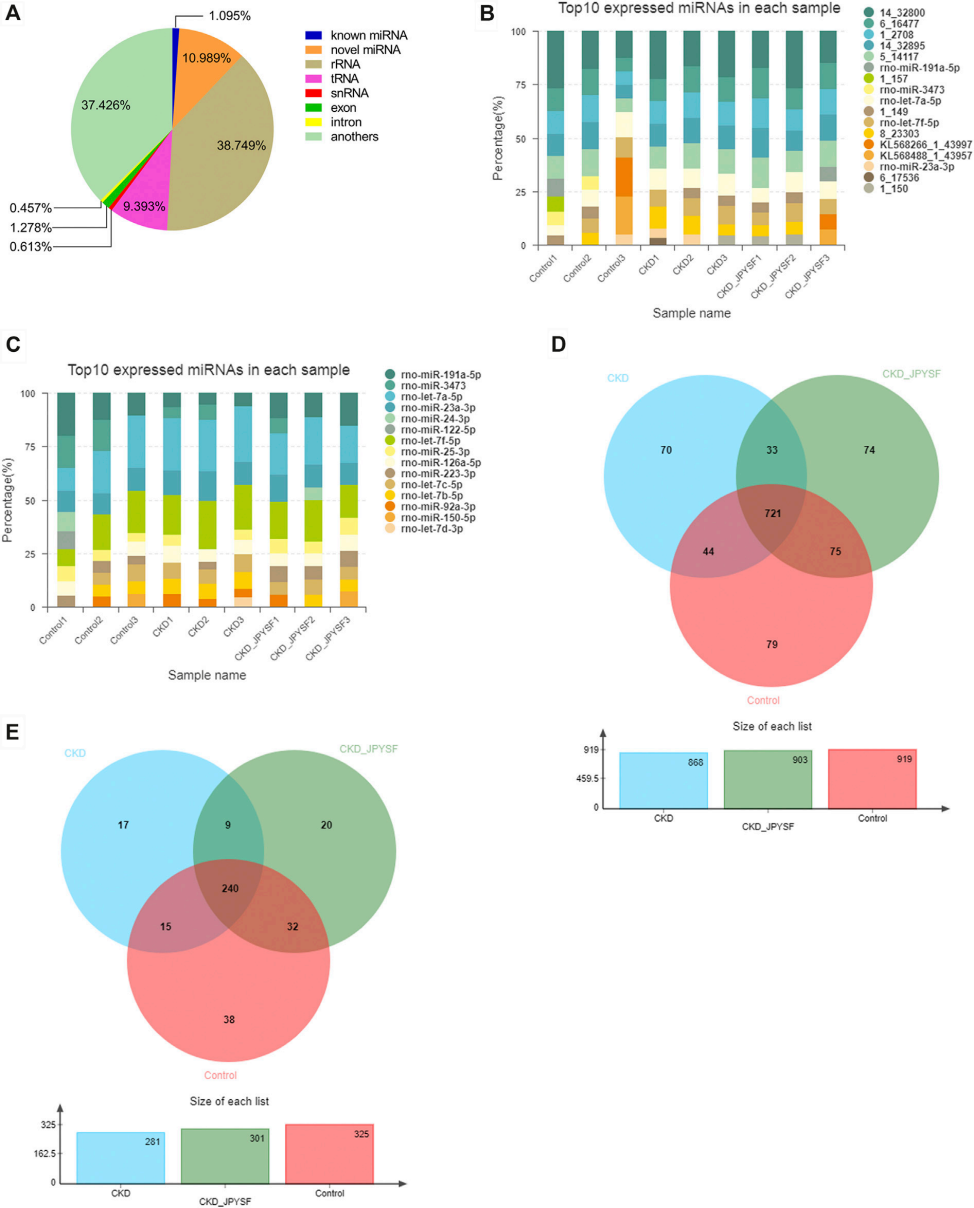
Overview of small RNA sequencing. **(A)** Proportion of various small RNAs. **(B)** Top 10 highest expressions of total miRNAs in each sample. **(C)** Top 10 highest expressions of known miRNAs in each sample. **(D)** Venn diagram analysis of total miRNAs in the three groups. **(E)** Venn diagram analysis of known miRNAs in the three groups.

A total of 104,123,785 raw reads and 102,018,331 clean reads were obtained from nine samples (3 samples per group). The average clean rate, Q20, and Q30 were 97.97%, 97.27%, and 93.62%, respectively, which indicated that the quality control of sequencing data was reliable (Table 3). In total, 40,480,280 RNA sequences were identified, including 443,328 known miRNA sequences (1.095%) and 4,448,389 novel miRNA sequences (10.898%) (Figure 3A). The top 10 highest expressions of total miRNAs and known miRNAs in each sample were shown in Figure 3B and Figure 3C, respectively. Venn diagram showed that 721 total miRNAs were shared in the control, CKD, and CKD + JPYSF group (Figure 3D), and 240 known miRNAs were commonly expressed in these three groups (Figure 3E).

**TABLE 3:** Basic statistical information on the small RNA sequencing data.

Sample	Raw reads	Clean reads	Clean rate (%)	Q20 (%)	Q30 (%)	GC (%)
Control 1	11845169	11763251	99.31	97.5	94	69.73
Control 2	11714568	11551257	98.61	97.46	93.86	66.84
Control 3	11335456	11075601	97.71	97.49	93.98	61.99
CKD 1	12075144	11854499	98.17	96.81	92.87	60.66
CKD 2	10149132	9884698	97.39	97.42	93.82	62.19
CKD 3	11739564	11381060	96.95	97.16	93.66	63.5
CKD + JPYSF 1	11611491	11356766	97.81	97.45	93.83	66.18
CKD + JPYSF 2	11879773	11621055	97.82	97.05	93.3	65.78
CKD + JPYSF 3	11773488	11530144	97.93	97.05	93.22	54.13

### Differentially Expressed miRNAs (DEMs) in Serum Exosomes

**FIGURE 4:**
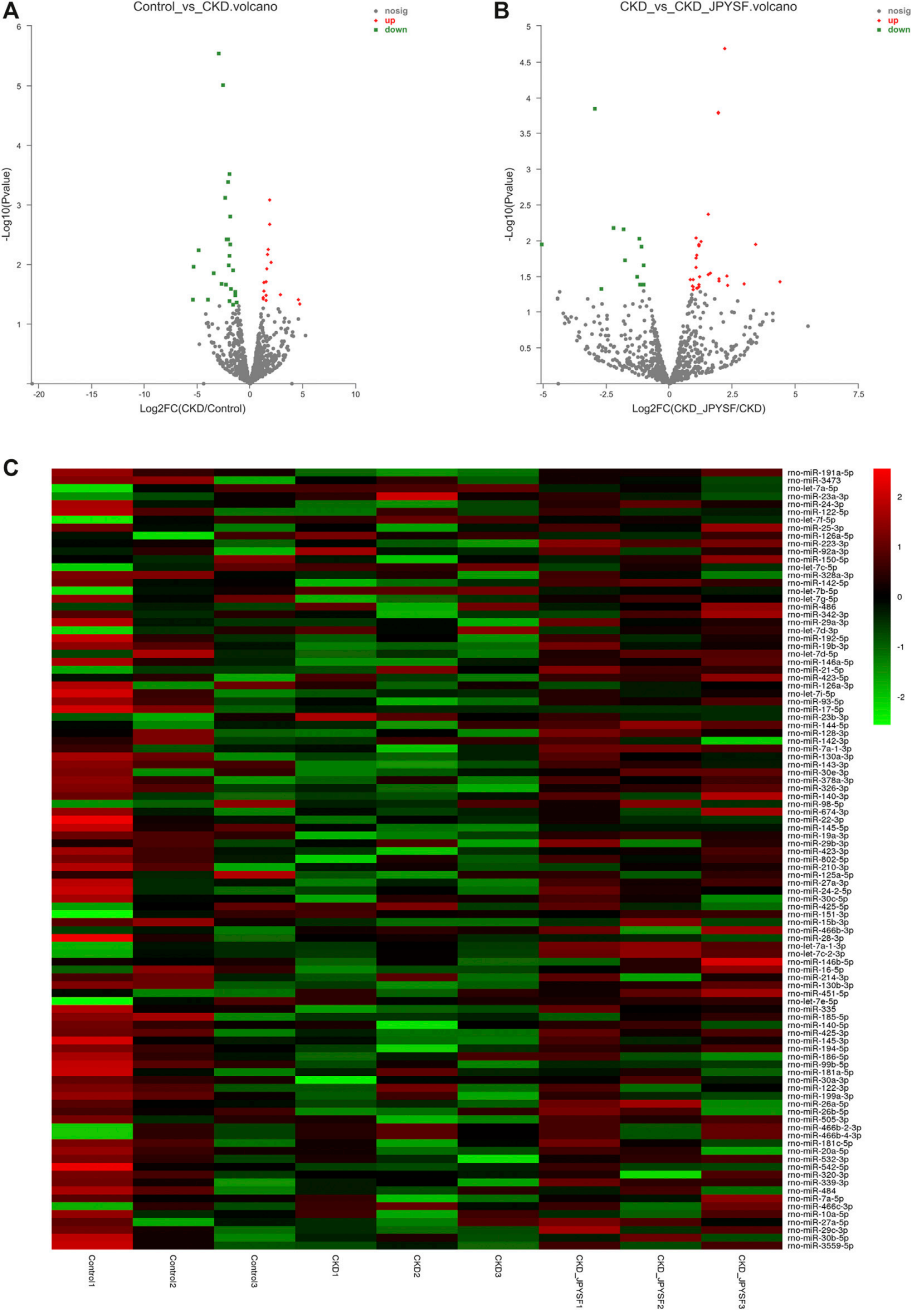
Analysis of differentially expressed miRNAs (DEMs) in serum exosomes. **(A)** Volcano plot analysis of DEMs between the control and CKD group. **(B)** Volcano plot analysis of DEMs between the CKD and CKD + JPYSF group. The green point represents downregulated miRNA; the grey point represents no significant miRNA; the red point represents upregulated miRNA. **(C)** Heatmap of hierarchical clustering analysis of top 50 highest expressions of known miRNAs in each sample. Green represents low expression; red represents high expression.

There were 43 miRNAs differentially expressed in serum exosomes of CKD rats compared with the control group (*p* value < 0.05 and |log_2_ fold change| ≥ 1, Supplementary Table S1). Among these miRNAs, 16 miRNAs were upregulated and 27 miRNAs were downregulated (Figure 4A). According to the same criteria, 47 DEMs were identified by comparing the CKD + JPYSF group with the CKD group (Supplementary Table S2). Among them, 34 miRNAs were upregulated and 13 miRNAs were downregulated (Figure 4B). Hierarchical cluster analysis showed the differences of top 50 highest expressions of known miRNAs in each sample (Figure 4C).

### Function and Pathway Analysis of the Target Genes of DEMs in Serum Exosomes

**FIGURE 5:**
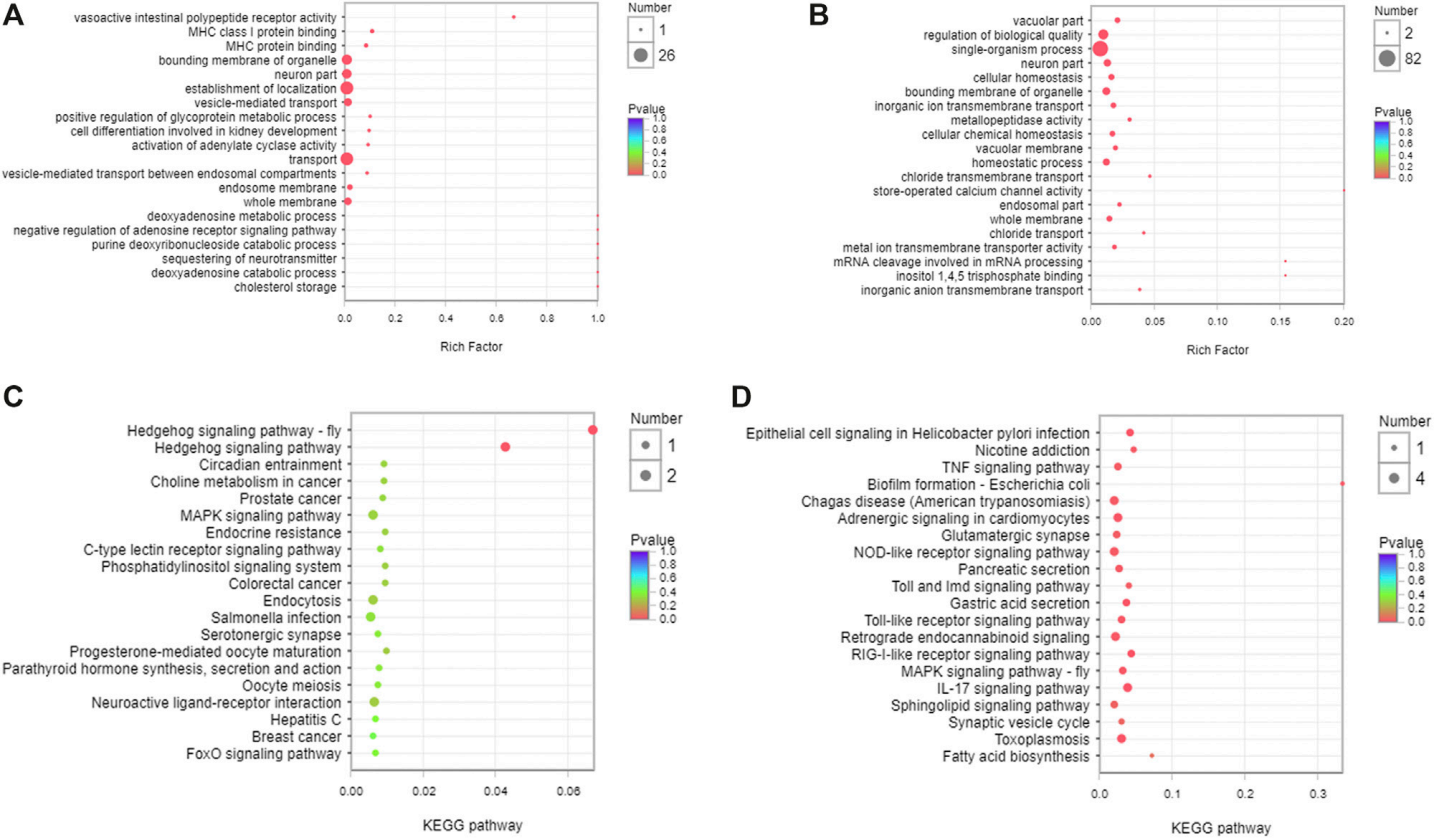
Function and pathway analysis of the target genes of DEMs in serum exosomes. **(A)** GO enrichment analysis of the target genes of DEMs between the control and CKD group. **(B)** GO enrichment analysis of the target genes of DEMs between the CKD and CKD + JPYSF group. **(C)** KEGG enrichment analysis of the target genes of DEMs between the control and CKD group. **(D)** KEGG enrichment analysis of the target genes of DEMs between the CKD and CKD + JPYSF group. The horizontal axis represents the enrichment rate, the size of the dot represents the number of genes, and the color of the dot represents *p* value.

GO and KEGG enrichment analyses were performed to identify the functions and mechanisms of DEMs target genes. The function and pathway of DEMs between the control and CKD group were mainly vasoactive intestinal polypeptide receptor activity and MHC class I protein binding in GO (Figure 5A) and Hedgehog signaling pathway in KEGG (Figure 5C). JPYSF treatment affected the function of chloride transport and inositol 1,4,5 trisphosphate binding in GO (Figure 5B) and nicotine addiction and RIG-I-like receptor signaling pathway in KEGG (Figure 5D).

### Screening and Validation of miRNAs That Respond to JPYSF Treatment

**FIGURE 6:**
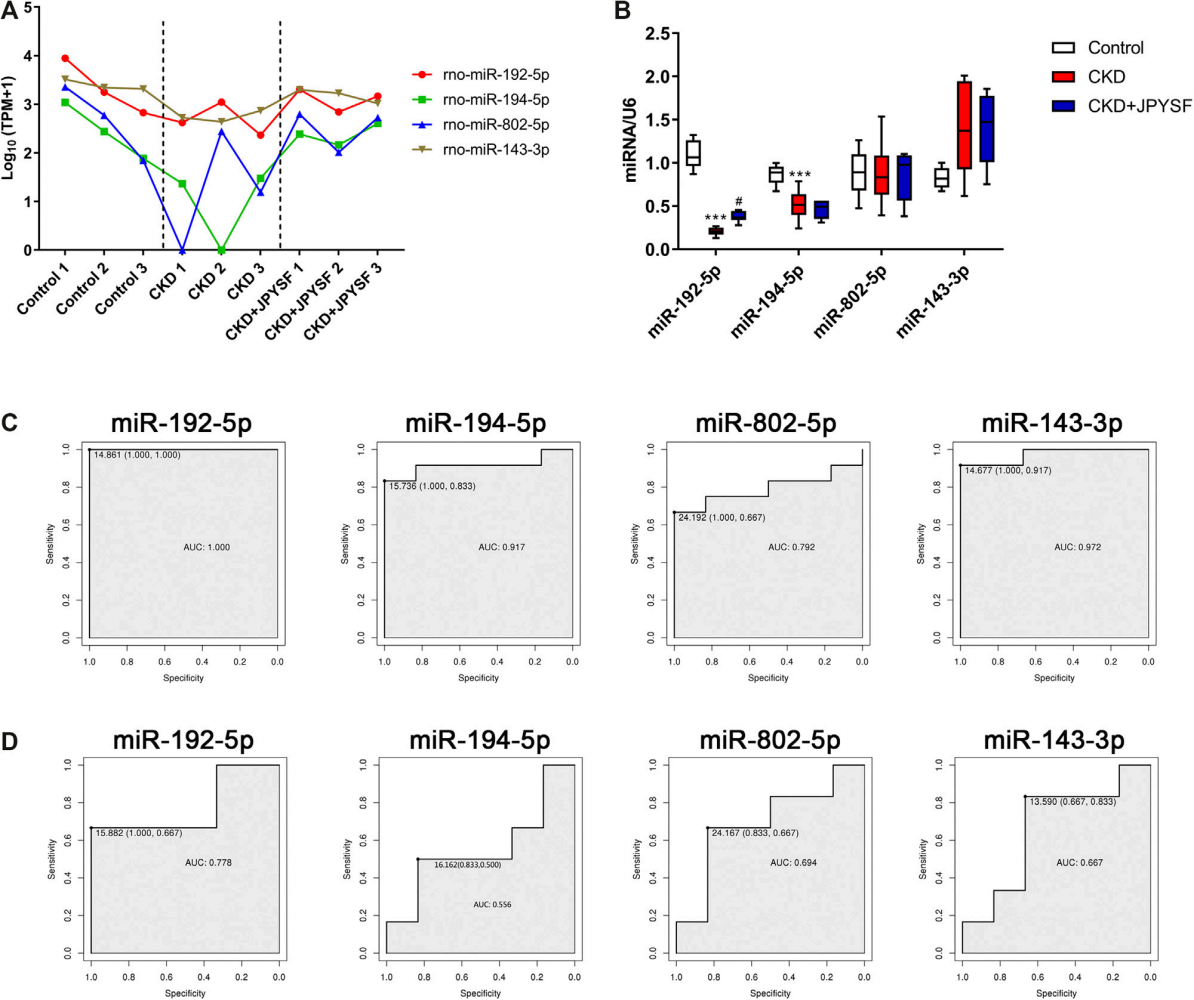
Screening and validation of miRNAs that respond to JPYSF treatment. **(A)** The expression levels of exosomal miR-192-5p, miR-194-5p, miR-802-5p, and miR-143-3p in each sample. The data was converted to transcripts per million (TPM) and expressed as Log_10_ (TPM+1). **(B)** The abundance of miR-192-5p, miR-194-5p, miR-802-5p, and miR-143-3p in the kidney tissue. n = 6 rats per group, ***P < 0.001 compared with the control group; ^#^ *P* < 0.05 compared with the CKD group. **(C)** ROC curves of miR-192-5p, miR-194-5p, miR-802-5p, and miR-143-3p for the diagnosis of CKD. **(D)** ROC curves of miR-192-5p, miR-194-5p, miR-802-5p, and miR-143-3p for distinguishing JPYSF treatment.

Four miRNAs (miR-192-5p, miR-194-5p, miR-802-5p, and miR-143-3p) were significantly downregulated in the CKD group and were markedly upregulated after JPYSF treatment (Table 4). The expression levels of these 4 miRNAs in each sample were shown in Figure 6A. To further identify the key miRNA involved in JPYSF treatment of CKD, the abundance of these 4 miRNAs in the kidney was tested by real-time PCR. The data indicated that only miR-192-5p was significantly downregulated in the CKD group and could be partially restored by JPYSF treatment (*p* < 0.05, Figure 6B). Moreover, receiver operating characteristic (ROC) analysis found that miR-192-5p was the most valuable miRNA for the diagnosis of CKD (area under the curve = 1.000, Figure 6C) and distinguishing JPYSF treatment (area under the curve = 0.778, Figure 6D). These data collectively suggested that miR-192-5p was the key miRNA involved in JPYSF treatment of CKD.

**TABLE 4:** Differentially expressed miRNAs responding to JPYSF treatment.

miRNA name	Fold change (CKD/Control)	Log_2_ fold change (CKD/Control)	*p* Value	Regulate	Fold change (CKD + JPYSF/CKD)	Log_2_ fold change (CKD + JPYSF/CKD)	*p* Value	Regulate
miR-192-5p	0.272	−1.879	0.005	Down	2.26	1.176	0.042	Up
miR-194-5p	0.094	−3.419	0.014	Down	10.62	3.409	0.011	Up
miR-802-5p	0.155	−2.694	0.021	Down	4.963	2.311	0.043	Up
miR-143-3p	0.262	−1.934	0.007	Down	2.253	1.172	0.045	Up

## Discussion

In the present study, we investigated the renoprotective effect and possible mechanism of JPYSF, a traditional Chinese herbal decoction, on adenine-induced CKD rat model. The results showed that JPYSF treatment significantly lowered Scr and BUN levels and alleviated renal pathological injury in CKD rats. Furthermore, serum exosomes were successfully isolated and identified. Small RNA sequencing revealed that 4 exosomal miRNAs (miR-192-5p, miR-194-5p, miR-802-5p, and miR-143-3p) were significantly downregulated in the CKD group and were markedly upregulated after JPYSF treatment. At last, miR-192-5p was identified as the most relevant miRNA for CKD diagnosis and JPYSF treatment.

Several lines of evidence indicated that exosome is involved in the renal physiology and pathologenic mechanisms of various kidney diseases including CKD (Thongboonkerd, 2019). Firstly, exosomes have a great potential for use as valuable diagnostic biomarkers in CKD. Exosomal miR-29c has been found to be correlated with both renal function and degree of histological fibrosis in CKD patients (Lv et al., 2013; Chun-Yan et al., 2018). Exosomal miR-181a was significantly decreased by about 200-fold in CKD patients compared to healthy controls (Khurana et al., 2017). Yu et al. included 38 CKD patients with different degrees of renal fibrosis and found that exosomal miR-200b was lower in the CKD group than in the normal group and decreased more significantly with fibrosis progression (Yu et al., 2018). However, Lange et al. reported that miR-21 was significantly upregulated in urinary exosomes of patients suffering from CKD (Lange et al., 2019). In addition to exosomal miRNAs, it was reported that urinary exosomal ceruloplasmin level was 10–20 times higher in CKD patients than in controls (Gudehithlu et al., 2019). The four exosomal miRNAs (miR-192-5p, miR-194-5p, miR-802-5p, and miR-143-3p) identified in this study have good diagnostic power for CKD, making them possible to become potential biomarkers. Secondly, exosomes are involved in renal fibrosis and further promote the progression of CKD. Furini et al. found that increased transglutaminase-2 (TG2) exosomal secretion by tubular epithelial cells lead to renal fibrosis in unilateral ureteric obstruction (UUO) mice (Furini et al., 2018). Another study using UUO model has indicated that transforming growth factor-β1 (TGF-β1) mRNA was transported by exosomes, which might activate fibroblast proliferation and development of renal fibrosis (Borges et al., 2013). Thirdly, exosomes can act as therapeutic intervention in CKD. Exosome miR-let7c derived from mesenchymal stem cells could attenuate renal fibrosis *in vivo* and *in vitro* (Wang et al., 2016).

In the present study, miR-192-5p was identified as the most relevant miRNA for CKD diagnosis and JPYSF treatment (Figure 6). miR-192 was one of the most abundant miRNAs in the kidney (Tian et al., 2008) and miR-192-5p targets the mRNA of the β1 subunit of the Na^+^/K^+^-ATPase (Mladinov et al., 2013). It has been reported that miR-192 may be a critical downstream mediator of TGF-β/Smad3 signaling in the development of renal fibrosis (Chung et al., 2010). The serum miR-192 level was negatively correlated with albuminuria and TGF-β1 expression in diabetic nephropathy (DN) patients (Ma et al., 2016). miR-192 prevented renal tubulointerstitial fibrosis in DN rat model by targeting early growth response factor 1 (Egr1) (Liu et al., 2018a). In IgA nephropathy (IgAN), lower serum exosomal miR-192 in IgAN patients correlated with more severe tubular atrophy, interstitial inflammation, and fibrotic tendency (Fan et al., 2019). In addition, urinary miR-192-5p was proposed as a potential biomarker of ischemia/reperfusion-induced kidney injury (Zou et al., 2017). Recently, miR-192-5p in the kidney was found to protect against the development of hypertension (Baker et al., 2019). The role and mechanism of miR-192-5p in CKD need further investigation.

There are few studies that tested the effects of Chinese herbal medicine on exosomes. Ruan et al. found that Suxiao Jiuxin pill, a TCM formula used for treating acute myocardial ischemia, could promote exosome secretion from mouse cardiac mesenchymal stem cells (Ruan et al., 2018). Scorpion and centipede are two anti-inflammatory Chinese medicines, which are used in the therapy of refractory asthma in China. By small RNA sequencing, bronchoalveolar lavage fluid exosomal miR-147-3p, miR-98-5p, and miR-10a-5p were found to be involved in alleviating asthma treated with scorpion and centipede (Tang et al., 2020). Although JPYSF has been used for decades to treat CKD in clinic, the mechanisms are still not fully elucidated. The present study indicated that miR-192-5p responded well to JPYSF’s treatment in CKD rats, which provides clue for further research.

## Conclusion

Taken together, the renoprotective effect of JPYSF on treating CKD was confirmed. Furthermore, small RNA sequencing revealed that serum exosomal miRNAs profiling was disturbed in CKD rats and could be modulated by JPYSF. These observations deepen our understanding of the therapeutic effects and mechanisms of JPYSF on CKD.

## Data Availability Statement

The datasets presented in this study can be found in online repositories. The names of the repository/repositories and accession number(s) can be found below: https://www.ncbi.nlm.nih.gov/, PRJNA680879.

## Ethics Statement

The animal study was reviewed and approved by Experimental Animal Ethics Committee of Guangzhou University of Chinese Medicine.

## Author Contributions

XL and SL conceived and designed the experiments. LZ and JL performed herbal preparation. SL, DL, SH, and FW carried out animal experiment and conducted the pathological analysis. BZ, YC, LZ, and JL contributed to data collection and manuscript review. XL and SL performed the experiments, analyzed the data, prepared figures, and wrote the manuscript. All authors have read and approved the manuscript.

## Funding

This study was supported by Natural Science Foundation of China (81973602 and 81804052), Shenzhen Science and Technology Plan Project (JCYJ20190812161001790, JSGG20191129102216637, JCYJ20180302173708520, and JCYJ20180507183842516), and Natural Science Foundation of Guangdong Province (2020A1515011151 and 2018A030313305).

## Conflict of Interest

The authors declare that the research was conducted in the absence of any commercial or financial relationships that could be construed as a potential conflict of interest.
