# Supplementary material for: Involvement of Circulating Exosomal MicroRNAs in Jian-Pi-Yi-Shen Formula Protection Against Adenine-Induced Chronic Kidney Disease
Source: Front Pharmacol. 2021 Feb 2;11:622658. doi: 10.3389/fphar.2020.622658 (PMC7884821; doi:10.3389/fphar.2020.622658)
Supplement: Supplementary file 1 [file datasheet1.pdf]

Supplementary Tables

Supplementary Table S1. Differentially expressed miRNAs (DEMs) in serum exosomes of the CKD group *versus* the control group

miRNA name	FC (CKD/Control)	Log2FC (CKD/Control)	*P* value	Regulate
KL568266.1_43997	0.129	-2.952251523	2.9E-06	down
KL568488.1_43957	0.129	-2.952251523	2.9E-06	down
7_19837	0.172	-2.536467007	9.81E-06	down
3_9674	0.262	-1.934076462	0.000303	down
20_41867	0.242	-2.047324364	0.000412	down
7_18293	0.242	-2.045678994	0.000419	down
7_19054	0.199	-2.326454866	0.00077	down
6_17536	3.662	1.872711187	0.000829	up
1_3880	0.273	-1.873208529	0.001599	down
5_14934	3.668	1.875106333	0.002127	up
9_24400	0.239	-2.062234025	0.003865	down
6_16930	0.218	-2.200734113	0.003888	down
rno-miR-192-5p	0.272	-1.878846147	0.004611	down
8_23303	3.319	1.730697435	0.005598	up
rno-miR-206-3p	0.035	-4.845352842	0.00584	down
6_16437	3.181	1.66964546	0.006865	up
rno-miR-143-3p	0.262	-1.934417183	0.007323	down
6_17616	4.08	2.028582356	0.009203	up
17_37945	0.25	-2.001098146	0.010372	down
rno-miR-1-3p	0.025	-5.332554988	0.010999	down
2_6480	3.072	1.619295007	0.012072	up
17_38055	0.326	-1.619270195	0.012513	down
rno-miR-194-5p	0.094	-3.418750478	0.014161	down
7_19237	2.928	1.550061674	0.019782	up
12_30286	2.539	1.34423134	0.020511	up
rno-miR-802-5p	0.155	-2.693564281	0.021493	down
rno-miR-335	0.202	-2.305475713	0.022257	down
2_6487	0.287	-1.802194244	0.026299	down
7_20238	2.493	1.318146198	0.027915	up
17_38196	0.374	-1.417421295	0.029012	down
13_31245	7.447	2.896651294	0.032489	up
3_9343	2.909	1.540442886	0.033409	up
4_11279	0.386	-1.372100713	0.033614	down

3_9625	2.387	1.255169679	0.036649	up
4_11255	2.388	1.255930397	0.038548	up
1_382	23.925	4.580434356	0.039358	up
18_39361	0.064	-3.960651357	0.039599	down
16_36177	0.024	-5.403649382	0.039722	down
11_28895	2.908	1.540245946	0.040503	up
7_20111	0.254	-1.976154368	0.041767	down
2_5834	0.418	-1.259805096	0.043564	down
9_23892	26.213	4.712218152	0.046267	up
1_4607	0.332	-1.590047132	0.048367	down

Supplementary Table S2. Differentially expressed miRNAs (DEMs) in serum exosomes of the CKD+JPYSF group *versus* the CKD group

miRNA name	FC (CKD+JPYSF/CKD)	Log2FC (CKD+JPYSF/CKD)	*P* value	Regulate
7_19837	4.573	2.193244	2.05E-05	up
4_10991	0.129	-2.95687	0.000145	down
4_11051	0.129	-2.95687	0.000145	down
20_41867	3.802	1.926936	0.000161	up
7_18293	3.796	1.924535	0.000165	up
7_19054	2.901	1.536621	0.004301	up
9_24948	0.214	-2.22564	0.006709	down
10_26533	0.283	-1.82055	0.007094	down
17_38055	2.079	1.055623	0.009325	up
7_19951	0.435	-1.20205	0.009557	down
3_9674	2.395	1.259841	0.010376	up
8_21713	2.252	1.171406	0.011368	up
8_21685	2.252	1.171406	0.011368	up
rno-miR-194-5p	10.62	3.408714	0.01137	up
5_15233	0.03	-5.05736	0.011523	down
8_23308	2.237	1.161654	0.011797	up
2_7329	2.264	1.178819	0.011882	up
8_23303	0.462	-1.11349	0.01223	down
2_5849	2.113	1.079485	0.01614	up
2_7421	2.061	1.043067	0.017894	up
18_39261	0.297	-1.75215	0.018966	down
6_16437	0.495	-1.01412	0.022179	down
2_6584	2.07	1.049381	0.023805	up
12_30357	3.105	1.634757	0.028889	up
rno-miR-130b-3p	2.908	1.539985	0.029962	up
9_25173	4.814	2.267298	0.031244	up
6_17616	0.41	-1.28601	0.032291	down
rno-miR-378a-3p	2.292	1.196344	0.032298	up
KL568488.1_43957	3.896	1.96203	0.034332	up
KL568266.1_43997	3.896	1.96203	0.034332	up
14_33202	1.789	0.839546	0.035526	up
14_33201	1.789	0.839546	0.035526	up
1_3880	1.935	0.952418	0.035649	up
rno-miR-6216	3.875	1.954145	0.036688	up
7_18949	20.765	4.376071	0.037542	up
rno-miR-532-5p	7.725	2.949592	0.040369	up

rno-miR-192-5p	2.26	1.176268	0.041759	up
8_21442	0.446	-1.1657	0.04197	down
7_19237	0.495	-1.01344	0.042063	down
rno-miR-802-5p	4.963	2.311204	0.0426	up
2_4973	1.899	0.925368	0.043592	up
1_1753	1.901	0.92713	0.04448	up
rno-miR-143-3p	2.253	1.172111	0.044649	up
rno-miR-326-3p	2.151	1.105274	0.04629	up
12_29860	2.101	1.070904	0.047194	up
3_10134	0.154	-2.69434	0.047668	down
rno-miR-142-5p	1.906	0.930227	0.049191	up
